# Flood Characteristics Drive River-Scale Macroplastic Deposition

**DOI:** 10.1021/acs.est.5c02969

**Published:** 2025-09-03

**Authors:** Louise J. Schreyers, Rahel Hauk, Nicholas Wallerstein, Adriaan J. Teuling, Remko Uijlenhoet, Martine van der Ploeg, Tim H. M. van Emmerik

**Affiliations:** † Hydrology and Environmental Hydraulics Group, 4508 Wageningen University, 6708 PB Wageningen, The Netherlands; ‡ Department of Water Management, Delft University of Technology, 2628 CN Delft, The Netherlands

**Keywords:** plastic, pollution, contaminants, hydrology, anthropocene

## Abstract

Plastic pollution is a global environmental challenge that negatively impacts species, ecosystems, and human livelihoods. River basins, with high population densities and poor waste management, are particularly exposed to plastic pollution. Floods amplify the presence of plastic in rivers by mobilizing previously deposited materials and introducing new plastics. Yet, the fate of these mobilized plastics remains unclear, with observations suggesting either downstream export or floodplain deposition. This study assesses flood impact on macroplastic deposition along river floodplains, using data from 14 events—five floods and nine nonflood conditions—across two Dutch rivers. Higher flood return periods increased macroplastic deposition, with the two largest floods depositing two to three times more macroplastic than nonflood conditions. Deposition mechanisms varied by flood type. Obstruction-based deposition dominated during an extreme summer flood, when macroplastics accumulated mainly in inundated vegetation. Low-energy deposition prevailed during a long winter flood, with high plastic concentrations found in wide floodplain sections where flow velocities decreased. Flood severity and plastic entry into the environment are both projected to increase. Therefore, we expect an even more prominent role for floods in the global distribution of plastic pollution.

## Graphical abstract

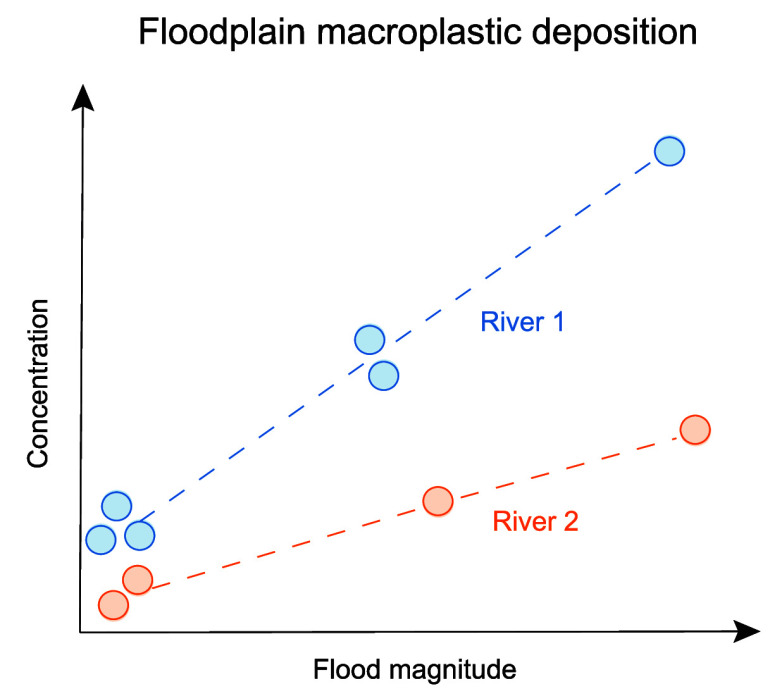

## Introduction

Plastic pollution is a global concern that poses risks to human health and contaminates ecosystems.^1^ Among the many forms of plastic pollution, macroplastics—defined as plastic items larger than 5 mm—represent a particularly visible and persistent problem in terrestrial and freshwater environments. Rivers are especially susceptible to macroplastic pollution due to their close connectivity with urban areas,^2^ which act as primary entry points for plastic pollution.^3^ In some cases, higher plastic concentrations were found in rivers than those observed in marine and coastal ecosystems.^4^

Macroplastics in rivers can cause various negative impacts, including ingestion by fauna^5^ and blockage of urban drainage infrastructure, which can lead to increased flood risk.^6^ Within rivers, riverbanks and floodplains are considered to be one of the largest sinks for plastic pollution, potentially storing more plastics than the river surface, water column, or riverbed sediments.^7,8^ However, the mechanisms controlling macroplastic deposition—particularly on floodplains—remain poorly understood. Recent research suggests that floods play a key role in driving macroplastic transport and deposition, in a manner similar to the behavior of inorganic sediments and large woody debris.^9–13^

Floods can cause significant damage to urban areas, leading to the influx of both waste and nonwaste plastic.^14^ Flooding of nonurbanized floodplains can also mobilize plastic deposited during previous high-flow events. In addition to increased plastic transport, overbank flows can result in substantial plastic deposition onto the floodplains. So far, flood-driven plastic deposition and transport have been documented for individual flood events, such as the summer 2021 flood along the Meuse river,^12,15^ the winter 2015–16 flood in Northwest England,^4^ and the winter 2018 flood in the Seine river in France.^16^ While these studies provide valuable insights into plastic mobilization and deposition, they do not fully address the variability of deposition across different flood events or the role of river and floodplain characteristics in shaping these processes. A comprehensive understanding of how different flood characteristics influence river-scale macroplastic deposition is missing. Flood characteristics such as hydrological type (fluvial, pluvial, coastal, and flash floods), duration, and magnitude can drive diverse transport mechanisms.^17^ Additionally, the factors that govern the spatial distribution of macroplastic deposition along floodplains remain largely unexplored, highlighting a critical gap in our understanding of river plastic pollution.

In this paper, we investigate macroplastic deposition across 14 events (including five floods), between 2018 and 2024, in the Rhine-Meuse delta. We quantified riverbank and floodplain macroplastic concentrations under both nonflood and flood conditions. Macroplastic concentrations were attributed to key deposition drivers, using a parsimonious modeling approach. We considered ten factors, including the characteristics of the river and floodplain and the proximity to potential plastic sources, based on literature. Our model assessed how strongly each factor predicted observed deposition patterns, offering new insights into floodplain retention of macroplastics.

## Methods

### Study Area Overview

We analyzed two Dutch rivers: the Meuse and the IJssel (Figure 1a,c). The Meuse is a rainfed river with a rapid hydrological response, a catchment of ∼33,000 km^2^ and a total length of 875 km, 260 km of which lie within the Netherlands.^18,19^ Originating in France, it flows through Belgium and Germany before entering the Netherlands. It has low summer flows and high winter flows, with ∼950 mm of evenly distributed annual precipitation.^20^ Its flow is regulated by weirs, canals, and withdrawals.

**Figure 1:**
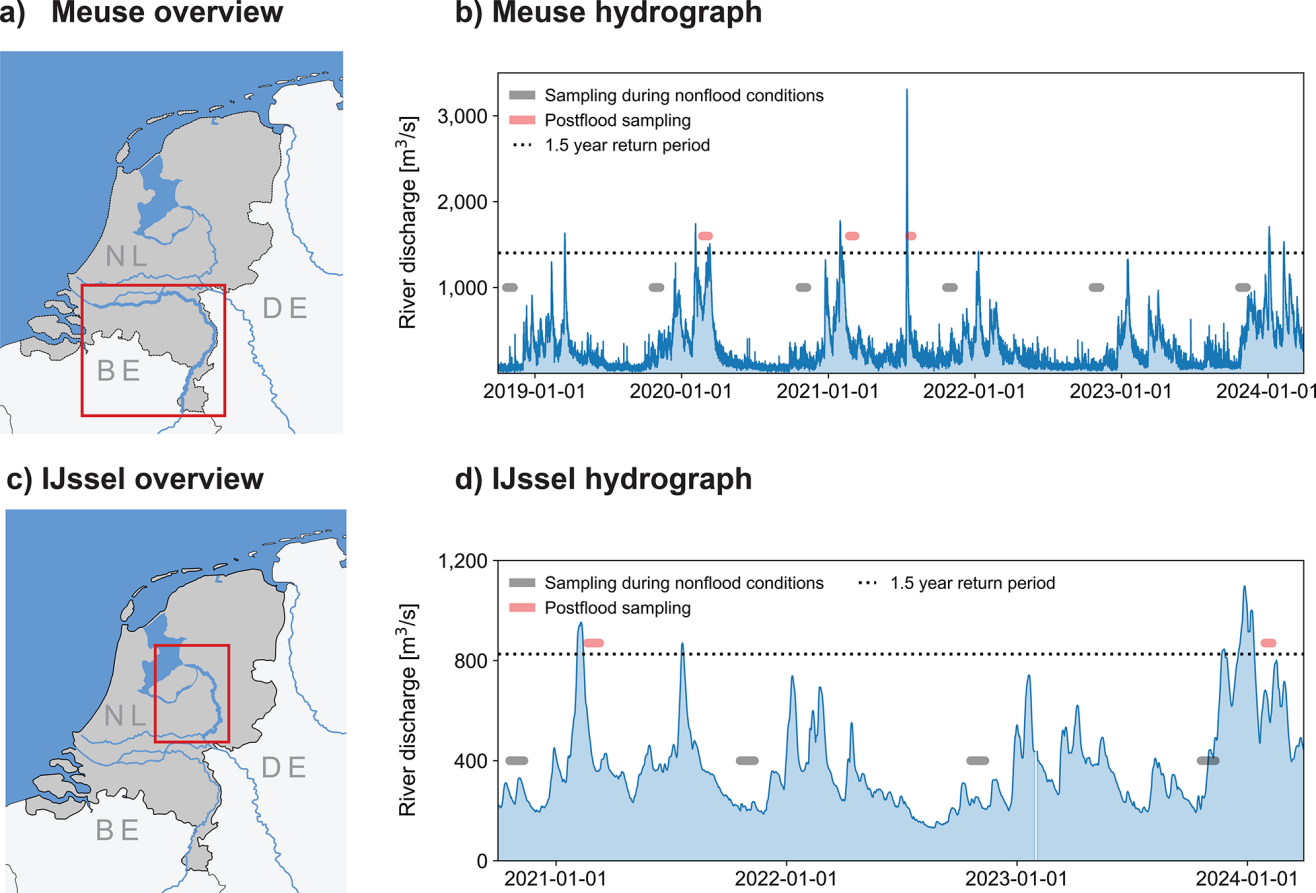
Localization maps and hydrographs of the Meuse and IJssel. (a) Overview of the Dutch Meuse. (b) Meuse hydrograph during macroplastic sampling periods. (c) Overview of the IJssel. (d) IJssel hydrograph during macroplastic sampling periods. The variations in height for the sampling periods are only for illustrative purposes.

The IJssel is a 120 km long distributary of the Rhine, which has a 185,260 km^2^ catchment and a mixed rain-snowmelt regime.^21^ Originating in the Swiss Alps, the Rhine flows through Germany and France before entering the Netherlands. Near the border, it splits into the Waal and Pannerden canal; the latter branches into the IJssel and Nederrijn. About 13–14% of the Rhine’s discharge at Lobith flows into the IJssel,^22^ which discharges into Lake IJssel near Kampen. IJssel water levels are driven by Rhine inflow, local rainfall, and storm surges in Lake IJssel.^23^

### Floodplain Macroplastic Observations

We used *Schone Rivieren* (English: Clean Rivers) data to quantify macroplastic concentrations during low-magnitude floods (winter 2020 and 2021) and nonflood conditions. Macroplastic concentrations on Dutch floodplains are monitored biannually by *Schone Rivieren* volunteers, using the River-OSPAR protocol.^24^ The sampled length, parallel to the waterline, is set at 100 m. The sampling width is defined by visible debris from recent high water and extends up to 25 m from the waterline. All visible litter items (>5 mm) are collected, counted, and categorized using the River-OSPAR classification,^24^ which includes 111 item categories. While the data include all anthropogenic macrolitter, 94% of the items found were macroplastic, so we refer to them as “macroplastic” in this study.

For the high-magnitude Meuse flood in summer 2021, we used the data set from Hauk et al.,^12^ collected between July 22 and August 4, 2021. We also carried out field sampling along the IJssel between January 25 and February 14, 2024, following the winter 2024 flood. Both campaigns were completed before cleanup operations (August 14, 2021 and February 15, 2024). Due to time constraints and limited accessibility, we applied a modified version of the River-OSPAR protocol. At each site, we sampled 2 m-long transects from the waterline. The transect width varied, extending up to the highest visible flood line. We aimed to sample three transects per site, but adjustments were made based on local conditions, like inundated areas or difficult terrain. In total, we sampled 25 sites along the Meuse and 23 sites along the IJssel. Each site corresponded to a single bank; when both banks were sampled, they were treated as separate sites. At each site, all visible macroplastics were counted and categorized according to the River-OSPAR protocol.

We converted macroplastic item count [#] to macroplastic mass [g] using data from a separate one-year sampling campaign at eight locations along Dutch riverbanks.^25^ In addition to counting and categorizing items following the River-OSPAR protocol, this study also weighed all 14,052 collected items. This allowed them to derive reliable mass statistics per item category. We refer to De Lange et al.^25^ for more information on the weighing protocol.

Macroplastic stocks were estimated by multiplying concentrations by floodplain areas. Uncertainties in these estimates arise from measurement limitations and item-to-mass conversion factors. Observer bias remains difficult to quantify due to the discrete item distribution.^26^ Residence times (Table 2) represent the estimated duration required for macroplastic stored in floodplains to be flushed out of the system. This was calculated as the ratio of stocks to annual transport. Macroplastic annual transport rates (15–41 tons/y for the IJssel and 56–75 tons/y for the Meuse) are based on annual floating macroplastic transport observations from a study by Van Emmerik et al.^27^ These floating transport rates were adjusted to total river transport, using empirical data from Schreyers et al.,^8^ which showed that approximately 70% of the total transported macroplastic mass remains at the river surface.

### Flood Severity Calculation

We considered five flood events: three on the Meuse and two on the IJssel (Figure 1b,d). River discharge data from the Dutch Directorate-General for Public Works and Water Management^28^ were used to estimate flood return periods with the Gumbel probability distribution^29^ and annual discharge maxima (Table S1). Discharge data from the Olst gauging station were used for the IJssel and from the Sint-Pieter station for the Meuse. Flood duration was defined as the period during which discharge exceeded the 1.5 year return period (Figure 1b,d), approximating bankfull discharge in natural rivers.^30–32^ For nonflood conditions (*T* < 1.5), a threshold-based approach was applied, fitting a generalized Pareto distribution (GPD).^33^

For the two higher-magnitude floods, flood severity was assessed along the river course (Figure 5). For the Meuse summer 2021 flood, severity was estimated from multiple gauging stations,^34^ showing flood attenuation downstream. For the winter 2024 IJssel flood, we estimated flood severity at Olst (the only discharge gauging station located along the IJssel, at km 68). For the upstream IJssel, flood severity was estimated based on discharge levels from the Lobith station (51.8619° N, 6.1186° E), using flow partitioning rates^22^ to distribute flow across the Waal, Nederrijn, and IJssel. Severity estimates based on Lobith and Olst data both indicate a ∼3 year return period, showing minimal variation in flood severity along the IJssel.

## Predictive Modeling of Macroplastic Concentrations

### Model Description

We developed a general modeling framework, using event-specific generalized linear models (GLMs).^35^ Similar to sediment and large wood deposition, we hypothesized that the longitudinal distribution of macroplastic along floodplains is likely influenced by the balance between supply and deposition factors, which determines floodplain capacity in retaining macroplastics.^36^

The model includes ten factors—independent of hydrological conditions—categorized into three groups: (i) floodplain characteristics (floodplain width, vegetation height, vegetation coverage index, lateral floodplain slope); (ii) river course characteristics (sinuosity index, river channel width, river channel slope), and (iii) proximity to potential sources (distance from upstream end of study area, distance from upstream wastewater treatment plant, distance from upstream tributary) (Figure 2). These factors were selected based on insights from research on plastic, sediment, and large wood and are expected to influence macroplastic deposition. Table 1 details the rationale for choosing these factors and their hypothesized response on deposition. Some factors may have nonuniform effects, with positive or negative impacts depending on conditions such as flow rates, morphology, and floodplain characteristics.

**Figure 2:**
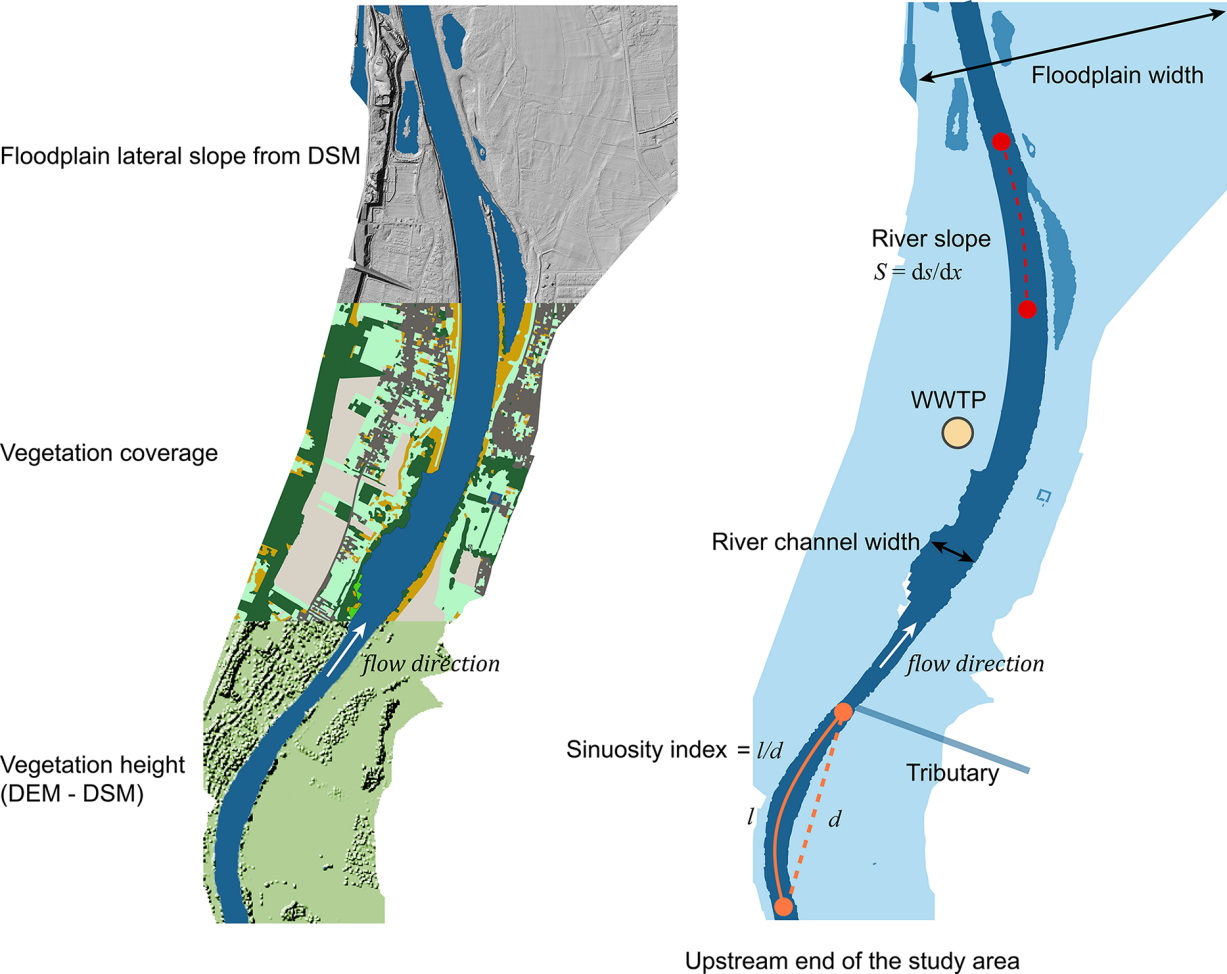
Schematic representation of the ten factors included in the model framework for simulating plastic concentrations. Note that specific model formulations often do not include all ten factors. The area represent the river channel and its floodplains. Here, d*s* represents the difference in water surface elevation, and d*x* represents the horizontal distance separating those two points.

**Table 1:** List of Variables Anticipated to Influence Macroplastic Deposition on Floodplains, Based on Available Literature on Plastic, Sediment, and Large Wood Deposition in River Systems^a^

Variable	Hypothesized response in deposition	Substantiation
Floodplain width	↑	Wider floodplains reduce cross-section averaged flow velocities,^37^ which in turn allows for greater deposition of plastic as the reduced energy of the water limits transport^38^
Floodplain vegetation height	↑	When the top vegetation height is lower than the inundation height, the vegetation acts as a physical barrier, trapping plastic, and promoting deposition^39^
	↓	When the vegetation height exceeds the inundation height, especially if only tree trunks are exposed to the flow, macroplastic items may bypass these features leading to reduced deposition or no noticeable effect
Floodplain vegetation coverage	↑	Greater vegetation coverage increases terrain roughness, which promotes the deposition of macroplastic^40^
Floodplain lateral slope	↓	Gentle slopes may reduce the velocity of overbank flows, promoting the settling of macroplastic, while steeper slopes could maintain higher flow velocities, reducing deposition
River channel sinuosity	↑	Larger sinuosity in the river’s channel increases water turbulence and mixing, which can lead to higher and lower flow velocities around the bends, favoring the settling and accumulation of macroplastic on adjacent floodplains^41^
River channel width	↓	Narrower channels are more likely to result in trapping of macroplastic on bank side obstructions as compared with wider channels carrying the same discharge^42^
River channel slope	↓	Increased river channel slope, indicative of stream power, increases the transport capacity of rivers^43^
Distance from upstream end of study area	↓	Gradual reduction in transport load as macroplastics move downstream, particularly if potential plastic sources are located upstream of the study domain
	↑	Proximity to river mouth can enhance macroplastic deposition due to tidal dynamics^44,45^
Distance from upstream WWTP	↓	WWTPs are point sources for plastic inputs into rivers^46^
Distance from upstream tributary	↓	Tributary inflow can lead to an increase in macroplastic concentrations in rivers, increasing the availability of macroplastics for deposition^47^
	↑	A clean tributary would actually dilute the macroplastic load of the main channel

a Each variable is accompanied by hypotheses indicating the expected direction of the relationship: an upward arrow denotes that an increase in the variable correlates with greater plastic deposition, while a downward arrow indicates the opposite effect. Additional substantiation regarding the mechanisms of macroplastic deposition on floodplains is also provided.

Floodplain characteristics were extracted by dividing the floodplain into 100 m sections along the river. Sections widths were determined using floodplain boundaries by the Ecotopen data set.^48^ Vegetation height was estimated by subtracting digital surface model (DSM) values from digital elevation model (DEM) values.^49,50^ The vegetation coverage was calculated using the Ecotopen vegetation classification. The floodplain lateral slope was also derived from the DSM. Since these three variables had two dimensions, values were averaged per 100 m section to ensure consistency with other variables. River channel width was similarly determined. Other variables were selected at different resolutions. The sinuosity index was estimated over 2 km segments to maintain resolution and avoid convergence toward unity, which can occur when calculated over very short segments. Channel slope was calculated as the gradient of water surface elevation (d*s*) over longitudinal distance (d*x*) (Figure 2); with water surface elevation derived from gauging stations.^28^ Major tributaries were manually selected, and the Waste Water Treatment Plant (WWTP) locations were extracted from *Stichting Nederlandse WaterSector*.^51^ All variables were documented per section, except in data-poor reaches between km 68 and 82 (Figure S2a).

### Model Performance

The models were initially fitted with all ten variables as linear terms. To improve the performance, we excluded variables with limited explanatory power and refitted some as exponential terms after observing poor fits with linear functions (results not shown). This iterative process optimized model performance by balancing complexity and fit. Performance was evaluated using the *R* ^2^ and the Akaike information criterion (AIC) values, where a lower AIC indicated better performance for models with similar *R* ^2^ values.^52^ Table S1 presents model formulations, performance metrics, and coefficients. For the Meuse summer 2021 event, model “1.j” was chosen as the best fitting model, while for the IJssel winter 2024 event, model “10.c” was selected.

To assess model robustness, we conducted a bootstrap analysis,^53^ using a “leave-one-out” cross-validation (LOOV) approach.^54^ This involves systematically removing one observation at a time, using the remaining *n* – 1 points to train the model, and testing performance on the excluded point. This process was repeated for all observations in the data set, allowing us to assess: (1) the robustness of the models between training and test subsets and (2) the uncertainty of the estimated coefficients. The test *R* ^2^ score was calculated by comparing predicted and actual values across all test iterations. The *R* ^2^ value across all test data for the IJssel winter 2024 event was 0.53, indicating moderate predictive capability. The Meuse summer 2021 event had a stronger median *R* ^2^ of 0.81. We calculated the relative interquartile range (IQR) of coefficients across all LOOV iterations, finding that all coefficients had a relative IQR below 0.1, indicating stability.

To evaluate the relative importance of each variable, we standardized both the predictor matrix *X* and the response variable *y* using a *z*-score transformation.^55^ This ensures that all variables are on the same scale. Standardized coefficients (Figure 4b,e) show the relative influence of each variable on the response.

## Results and Discussion

### Floodplain Macroplastic Deposition Increases with Flood Severity

Macroplastic deposition on floodplains increases with flood severity, defined by the flood’s return period. Higher-magnitude floods lead to increased macroplastic mass concentrations on floodplains than lower-magnitude events and nonflood conditions (Figure 3). This trend is supported by strong correlations between macroplastic mass concentrations and both flood return period (*T*) (Spearman’s ρ = 0.52, Pearson’s ρ = 0.65, *p*-value <0.05) and river discharge (Spearman’s ρ = 0.76, Pearson’s ρ = 0.84, *p*-value <0.05). The most severe flood (*T* > 100 years) on the Meuse resulted in the highest macroplastic mass concentrations, with 11.2 g/m^2^, more than twice that of nonflood conditions (5.0 g/m^2^). Similarly, the largest flood event on the IJssel in winter 2024 (*T* = 3 years) led to the highest recorded macroplastic mass concentrations for that river (3.3 g/m^2^), about three times more than during nonflood conditions.

**Figure 3:**
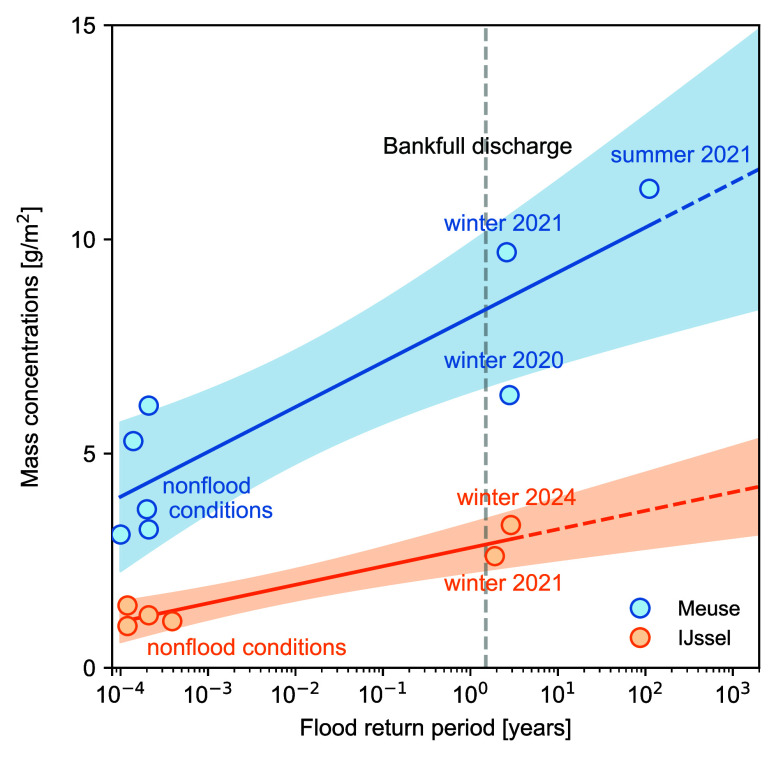
Observed increase in macroplastic mass concentrations as a function of flood return period. Bankfull discharge is indicated using the 1.5 year return period, consistent with literature for natural rivers that are in equilibrium.^30–32^ The shaded areas represent the 95% confidence interval. The dashed line projects observed trends.

This relationship resembles trends in sediment studies, where higher floodplain deposition rates correspond to increased flood severity.^9,56^ Differences in regression intercepts between the Meuse and IJssel indicate that the relationship is river-specific, likely reflecting baseline plastic pollution levels (Figure 3). Although deposition generally increases with flood severity, the Meuse shows significant variability between events. Notably, some nonflood periods exceeded winter 2020 flood concentrations, suggesting that factors beyond flood severity, such as legacy plastics or postflood cleanup efforts, may also influence macroplastic concentrations. Furthermore, the relationship between macroplastic item concentrations and return period (Figure S1) is less straightforward than that of mass concentrations. This discrepancy may be attributed to fragmentation processes,^57^ where item numbers increase without a corresponding mass gain.

The two highest-magnitude floods deposited 4620 tons of macroplastic along the 240 km of the Dutch Meuse, and 610 tons of macroplastic along 120 km of the IJssel (Table 2). Postflood macroplastic stocks were two to three times higher than those estimated during nonflood conditions. Comparing these stock values with upstream and downstream annual in-river macroplastic transport reveals that the total macroplastic mass retained during floods equates to 62–83 years of annual transport for the Meuse and 15–41 years for the IJssel (Table 2). These residence times indicate how long it would take for the river to transport an equivalent mass of macroplastic to that retained on banks and floodplains. While not precise due to uncertainties, these values align with evidence of multidecadal macroplastic accumulation on floodplains.^58^

**Table 2:** Floodplain and Riverbank Macroplastic Stocks Increase Significantly Following Major Floods^a^

	Annual transport [tons/y]	Floodplain/riverbank stocks [tons]		Residence times [y]	
		Flood	Nonflood	Flood	Nonflood
Meuse	56–75	4620	1937	62–83	26–35
IJssel	15–41	610	222	15–41	5–15

a Non-flood stock values represent the average from ten events. Annual macroplastic transport rates are derived from literature.^8,27^ Details on the calculation of these metrics are provided in the subsection “Floodplain Macroplastic Observations”.

### Flood Conditions Govern Spatial Patterns and Drivers of Macroplastic Deposition

We accurately estimated macroplastic concentrations for the two highest magnitude floods (*R* ^2^ = 0.93 for the Meuse summer 2021 flood and *R* ^2^ = 0.83 for the IJssel winter 2024 flood, Figure 4b,f), using models based on eight factors. For the Meuse flood, the primary governing factor was the distance from upstream study boundary (Figure 4b). This is consistent with extensive damage to the built environment in the Belgian Meuse, particularly in the Vesdre tributary.^59,60^ Large quantities of macroplastics were likely mobilized but not transported far due to the localized deposition mechanisms such as debris trapping in vegetation. We hypothesize that macroplastics originating from these heavily damaged areas were deposited near their sources, explaining high upstream concentrations in the Dutch Meuse. This pattern may also reflect flood severity, which was highest upstream (return period >100 years at km 10) and lower downstream (∼10 years at km 155),^34^ increasing the likelihood of macroplastic mobilization and deposition near source zones.

**Figure 4:**
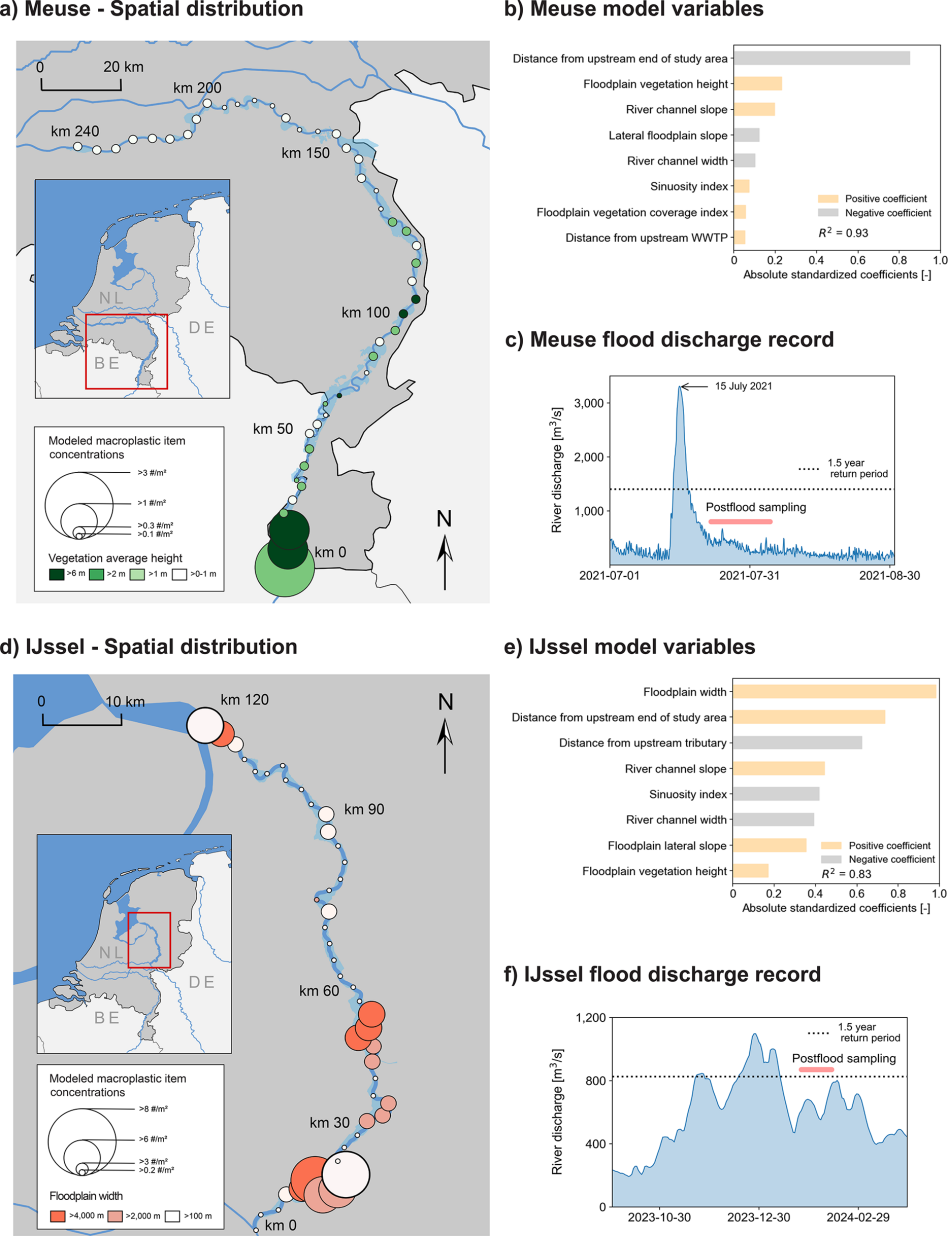
Modeled macroplastic concentrations along the Meuse (a) and IJssel (d) rivers, showing the impact of key explanatory variables. Concentration values were aggregated in bins of 5 km for the Meuse and 2.5 km for the IJssel. For both floods, eight variables significantly explained macroplastic deposition (b,e). The hydrological characteristics of the floods differed: the summer 2021 Meuse flood was an extreme event (c), whereas the winter 2024 IJssel flood was a long winter flood event (f).

We characterize the summer 2021 Meuse flood as following an obstruction-based deposition pattern, with most macroplastic deposited in vegetated floodplains (Figure 5a). These high accumulation zones coincide with steeper river slopes (∼0.04 m/m vs ∼0.01 m/m elsewhere along the Dutch Meuse) (Figure 4b) and flow velocities up to 6 m/s.^19^ Macroplastic concentrations dropped rapidly after km 15 (Figure 4a), likely due to upstream retention by vegetation. This highlights the role of riparian vegetation in trapping macroplastic during high-energy flow conditions^61^ and reducing its downstream transport.

**Figure 5:**
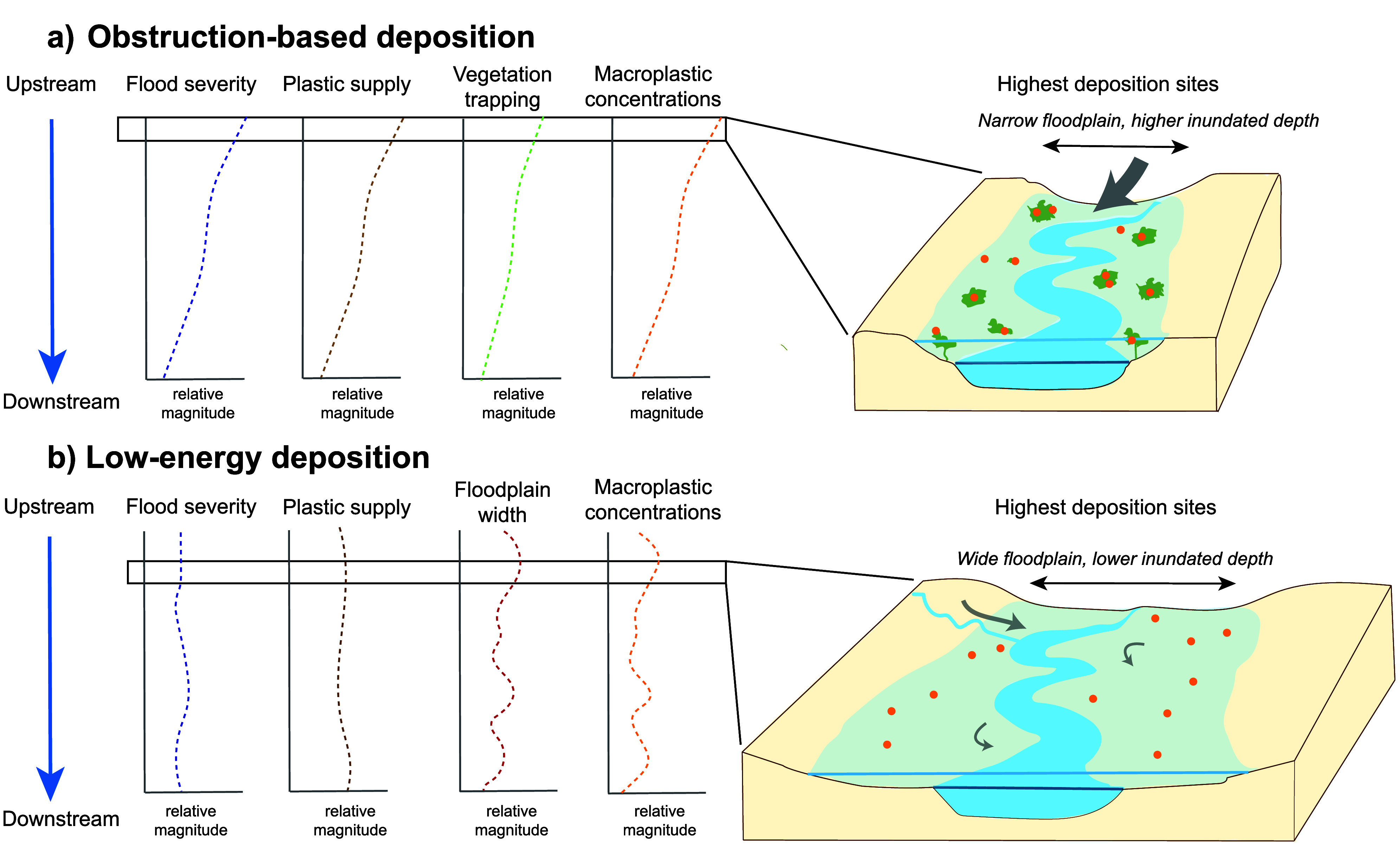
Conceptual representation of macroplastic deposition patterns along floodplains. (a) Obstruction-driven deposition, observed during the summer 2021 Meuse flood. The main macroplastic supply source (gray arrow) originates from the upstream end of the study domain, with macroplastics primarily depositing on floodplain zones with inundated trees, deep floodplain water levels, and high flow velocities. (b) Low-energy deposition observed during the winter 2024 IJssel flood. Macroplastic supply sources are more diffuse, and greater deposition rates are observed in wide floodplains with reduced cross-sectional flow velocities. The schematized trends are conceptual illustrations derived from our observations and predictive modeling, and relevant literature (cf. section “Flood Severity Calculation”). As such, further research is needed to validate and refine this conceptual representation.

The winter 2024 IJssel flood followed a different pattern, with floodplain width as the primary driver of macroplastic deposition (Figure 4d). Wider floodplains correspond to higher concentrations, likely due to reduced cross-sectional flow velocities.^37,38^ Proximity to tributaries was also important (Figure 4e), consistent with short macroplastic transport distances (0.2–12 km/day).^44,58,60^ Unlike the 2021 flood, this event caused little damage to the built environment. The observed macroplastic increase likely reflects mobilization of plastic buried in the riverbed or in suspension. We classify this event as a low-energy deposition pattern (Figure 5b).

The model performance was lower during nonflood and low-magnitude flood conditions (*R* ^2^ < 0.5) (Table S2). A strong correlation between return period and model accuracy (Pearson’s ρ = 0.72; Spearman’s ρ = 0.67; *p* < 0.05) suggests improved performance during larger floods. During lower-magnitude events, floodplains were inactive or only partially flooded, limiting the influence of floodplain-related variables. Point-source variables also showed no significant correlation with macroplastic concentrations during low-magnitude floods and nonflood conditions, suggesting these sources were then inactive.^62^ This aligns with previous findings by Roebroek et al.,^26^ who found that macroplastic deposition during nonflood conditions was not significantly influenced by factors such as wind speed, precipitation, or water levels.

### Impact of Extreme Floods on Macroplastic Deposition

The summer 2021 flood deposited 4620 tons of macroplastic in the Dutch Meuse floodplains—nearly 30% of the catchment’s annual mismanaged plastic waste (15,915 tons/y).^63^ This high deposition is due to the large mass per item (mean: 13.4 g/#), four times higher than that during the IJssel 2024 flood (3.3 g/#) and higher than other rivers globally, such as the Saigon (3.2 g/#).^64^ Macroplastic concentrations ranged from 0.4 to 184 g/m^2^ (Figure S2a). The high concentrations upstream of the Dutch Meuse likely reflect extensive flood damage to the built environment^59^ and inputs from combined sewer overflows (CSOs).^12^ Deposited material included both waste and nonwaste macroplastics mobilized during the flood.^14^ This highlights the role of extreme floods in amplifying plastic pollution beyond routine leakage from water infrastructure. Addressing this issue thus requires not only waste management but also flood resilience, to reduce both damage to built environments and plastic mobilization.^17^

Our results show strong spatial variability in macroplastic deposition along the Dutch Meuse, with concentrations decreasing exponentially from upstream to downstream. Due to access and safety constraints, we could not sample the most affected areas, such as the Belgian Meuse and tributaries. It is likely that even higher concentrations occurred in those areas as dense debris carpets at the water surface were documented.^65^ Consequently, our estimates likely under-represent the total macroplastic deposition along the Meuse, especially upstream. Our analysis is limited to the Dutch Meuse and does not capture the full river corridor.

Plastic entry into aquatic systems is expected to rise with increasing global plastic production and consumption.^66^ In addition, climate change may lead to more frequent severe floods in certain regions of the world.^67,68^ For instance, the return period of 20 year floods is projected to decrease from the late 20th and late 21st centuries, meaning such events will occur more frequently.^69^ Since 24% of the global population lives in flood-prone areas,^70^ flood-driven plastic mobilization is likely to increase. Strengthening flood resilience could mitigate damage to built environments and reduce plastic inputs during extreme events. Inadequate floodplain cleanup may contribute to a growing legacy of macroplastic on floodplains from past deposition events. Long retention times of macroplastic on floodplains^58^ increase the potential for biochemical fragmentation through photo-oxidation,^57^ increasing risks of ingestion by fauna.

### Toward Prediction of Flood-Driven Macroplastic Deposition: Knowledge Gaps and Future Directions

Our study highlights the significant impact of floods on macroplastic deposition in floodplains. While floods increase riverine macroplastic transport,^15^ this does not necessarily translate to greater export to coastal areas. Indeed, substantial deposition can occur along the river course, on riverbanks and floodplains. The extent to which macroplastics are transported or retained depends on locations and may vary over the course of a flood event as shifting flow conditions dynamically influence the balance between transport and deposition.

Our research is limited to two lowland rivers in the Netherlands with relatively low baseline pollution^71^ and covers a small number of flood events (*n* = 5). Nevertheless, this represents a larger data set of flood-related plastic deposition than any previous study, providing unprecedented insights into the role of floods in macroplastic deposition. Future studies should include a broader range of events, river systems, and pollution levels. In situ sampling during and after floods is crucial but challenging: continuous hydrological monitoring is necessary for safety and access. In addition, our model could be improved by including variables like combined sewer outfalls; proximity to flood-damaged or urban infrastructure as these are known plastic sources during floods.^60,72^

Literature suggests the existence of multiple flood-transport regimes for debris,^73^ potentially leading to distinct deposition patterns.^74^ For instance, flash floods in small systems may result in catchment-wide flushing,^75^ a pattern we did not observe in our study. Previous research^4^ reported a decrease in microplastic abundance in riverbed sediments following floods, contrasting with our findings of increased macroplastic deposition on floodplains. Hauk et al.^12^ also observed that certain plastic types were preferentially deposited, while others were flushed out. These elements show the complexity of river plastic transport and retention during floods, where both flushing and retention processes can coexist depending on flood dynamics, river morphology, river sinks, and plastic characteristics.

To effectively reduce macroplastic deposition on floodplains, further studies are needed on the spatial distribution to inform mitigation. Our typologies of floodplain macroplastic deposition (Figure 5) suggest distinct spatial patterns. Macroplastics deposited during flood events with obstruction-based deposition patterns clusters around or within riparian vegetation. In contrast, macroplastics deposited during low-energy deposition might be distributed in lines parallel to the high water line.^76^ Identifying these patterns can support targeted, cost-effective interventions. Our model, applicable to other flood events, does not rely on hydrological conditions but requires activated floodplains as floodplain width and vegetation height are key to explaining deposition patterns.

## Supplementary Material



## Data Availability

The data used in this study is made publicly available at: 10.4121/19415a1b-1f4e-4c5d-acc3-69545804698c.

## Notes

Flood event characteristics; model formulations, coefficients, and performance; macroplastic item concentrations as a function of flood return period; and modeled macroplastic concentrations along the upstream-downstream gradient (PDF)
