# Supplementary material for: Flood Characteristics Drive River-Scale Macroplastic Deposition
Source: Environ Sci Technol. 2025 Sep 3;59(36):19414–23. doi: 10.1021/acs.est.5c02969 (PMC12444984; doi:10.1021/acs.est.5c02969)
Supplement: Supplementary file 1 [file es5c02969_si_001.pdf]

Supporting Information for 'Flood characteristics drive river-scale macroplastic deposition'

Louise J. Schreyers,*,† Rahel Hauk,† Nicholas Wallerstein,† Adriaan J. Teuling,† Remko Uijlenhoet,†,‡ Martine van der Ploeg,† and Tim H.M. van Emmerik†

†*Hydrology and Environmental Hydraulics Group, Wageningen University, 6708 PB, Wageningen, The Netherlands*

‡*Department of Water Management, Delft University of Technology, 2628 CN, Delft, The Netherlands*

E-mail: *l.schreyers@gmail.com

Summary: 5 pages, 2 tables, 2 figures.

Table S1: Flood event characteristics. 'Summer' and 'winter' are used for brevity to indicate the general season of the flood events. Note that events can span multiple months; for example, 'winter 2024' refers to the event that occurred in December 2023–January 2024.

River	Flood event	Maximum river discharge [m^3/s]	Flood duration [d]	Flood return period [y]	Time since previous 1.5-year flood [d]
Meuse	summer 2021	3,310	3.3	111	147
	winter 2021	1,775	3.3	2.8	264
	winter 2020	1,742	4.8	2.6	229
IJssel	winter 2024	1,097	30.0	2.9	830
	winter 2021	804	10.0	1.9	266

Table S2: Model formulations, coefficients and performance. Coefficients marked with an asterisk (*) indicate an exponential transformation. Different formulations for the same event are grouped under the same model number, with variations indicated by the letters (e.g., model 1.a and 1.b represent different formulations for event 1).

		**Floodplain characteristics**				**River channel characteristics**			**Point sources**			
**Model**	**Calibrated dataset**	**Width**	**Vegetation height**	**Vegetation coverage index**	**Lateral slope**	**Sinuosity**	**Width**	**Slope**	**Distance from upstream end of study area**	**Distance from upstream WWTP**	**Distance from upstream tributary**	**R^2 (AIC)**
1.a	Meuse summer 2021			$3.6 \cdot 10^{-2}$ (*)	$2.6 \cdot 10^{-2}$		$3.5 \cdot 10^{-4}$	$2.7 \cdot 10^{0}$	$4.8 \cdot 10^{0}$ (*)			0.92 (-75)
1.b	Meuse summer 2021	$-4.5 \cdot 10^{-4}$	$1.8 \cdot 10^{-1}$	$5.4 \cdot 10^{-1}$	$1.1 \cdot 10^{-2}$	$-1.5 \cdot 10^{0}$	$-2.8 \cdot 10^{-3}$	$9.0 \cdot 10^{1}$	$1.0 \cdot 10^{-5}$	$-2.0 \cdot 10^{-5}$	$4.0 \cdot 10^{-5}$	0.73 (-19)
1.c	Meuse summer 2021	$-7.0 \cdot 10^{-5}$	$1.4 \cdot 10^{-1}$	$-1.6 \cdot 10^{-1}$	$3.4 \cdot 10^{-2}$	$1.2 \cdot 10^{0}$	$-3.5 \cdot 10^{-3}$	$-2.2 \cdot 10^{1}$	$5.4 \cdot 10^{0}$ (*)	$1.0 \cdot 10^{-5}$	$-1.0 \cdot 10^{-5}$	0.93 (-72)
1.d	Meuse summer 2021		$1.2 \cdot 10^{-1}$	$-7.9 \cdot 10^{-2}$	$3.1 \cdot 10^{-2}$	$7.6 \cdot 10^{-1}$	$-2.8 \cdot 10^{-3}$	$-1.3 \cdot 10^{1}$	$5.2 \cdot 10^{0}$ (*)			0.92 (-76)
1.e	Meuse summer 2021		$1.1 \cdot 10^{-1}$		$2.8 \cdot 10^{-2}$	$6.3 \cdot 10^{-1}$	$-2.5 \cdot 10^{-3}$	$-1.3 \cdot 10^{1}$	$5.1 \cdot 10^{0}$ (*)			0.92 (-78)
1.f	Meuse summer 2021		$1.0 \cdot 10^{-1}$		$4.0 \cdot 10^{-2}$		$3.3 \cdot 10^{-4}$	$1.0 \cdot 10^{0}$	$4.8 \cdot 10^{0}$ (*)			0.92 (-76)
1.g	Meuse summer 2021		$1.9 \cdot 10^{-1}$						$4.9 \cdot 10^{0}$ (*)			0.85 (-59)
1.h	Meuse summer 2021	$-3.4 \cdot 10^{-4}$	$1.7 \cdot 10^{-1}$	$4.2 \cdot 10^{-1}$	$2.7 \cdot 10^{-2}$	$-8.8 \cdot 10^{-1}$	$1.7 \cdot 10^{-3}$	$5.4 \cdot 10^{1}$		$-2.0 \cdot 10^{-5}$	$3.0 \cdot 10^{-5}$	0.72 (-19)
1.i	Meuse summer 2021		$1.4 \cdot 10^{-1}$	$-1.4 \cdot 10^{-1}$	$3.7 \cdot 10^{-2}$	$1.0 \cdot 10^{0}$	$-3.2 \cdot 10^{-3}$	$-2.5 \cdot 10^{1}$	$5.5 \cdot 10^{0}$ (*)	$1.0 \cdot 10^{-5}$	$-1.0 \cdot 10^{-5}$	0.93 (-74)
1.j	Meuse summer 2021		$1.3 \cdot 10^{-1}$	$-8.1 \cdot 10^{-2}$	$3.2 \cdot 10^{-2}$	$7.8 \cdot 10^{-1}$	$-3.1 \cdot 10^{-3}$	$-1.8 \cdot 10^{1}$	$5.3 \cdot 10^{0}$ (*)	$1.0 \cdot 10^{-5}$		0.93 (-75)
2.a	Meuse winter 2021	$2.6 \cdot 10^{-6}$	$1.8 \cdot 10^{-1}$	$6.9 \cdot 10^{-2}$	$-3.7 \cdot 10^{-2}$	$-2.1 \cdot 10^{-1}$	$5.3 \cdot 10^{-3}$	$7.3 \cdot 10^{-1}$	$-1.8 \cdot 10^{-6}$	$5.5 \cdot 10^{-6}$	$-1.1 \cdot 10^{-5}$	0.47 (-43)
3.a	Meuse winter 2020	$-1.4 \cdot 10^{-4}$	$4.0 \cdot 10^{-3}$	$1.1 \cdot 10^{-1}$	$6.7 \cdot 10^{-2}$	$2.4 \cdot 10^{0}$	$-8.4 \cdot 10^{-3}$	$-3.3 \cdot 10^{1}$	$-7.8 \cdot 10^{-6}$	$-8.1 \cdot 10^{-6}$	$3.9 \cdot 10^{-5}$	0.43 (-13)
4.a	Meuse fall 2018	$-1.1 \cdot 10^{-5}$	$3.5 \cdot 10^{-3}$	$-3.8 \cdot 10^{-3}$	$9.0 \cdot 10^{-4}$	$-5.0 \cdot 10^{-3}$	$-4.8 \cdot 10^{-4}$	$3.7 \cdot 10^{0}$	$6.0 \cdot 10^{-7}$	$5.6 \cdot 10^{-6}$	$-1.4 \cdot 10^{-6}$	0.24 (-127)
5.a	Meuse fall 2019	$2.5 \cdot 10^{-5}$	$-1.7 \cdot 10^{-2}$	$-4.3 \cdot 10^{-2}$	$2.0 \cdot 10^{-2}$	$1.8 \cdot 10^{-1}$	$2.2 \cdot 10^{-4}$	$-3.2 \cdot 10^{0}$	$-1.2 \cdot 10^{-6}$	$-8.0 \cdot 10^{-7}$	$7.1 \cdot 10^{-6}$	0.23 (-151)
6.a	Meuse fall 2020	$-2.6 \cdot 10^{-5}$	$7.7 \cdot 10^{-2}$	$-7.2 \cdot 10^{-2}$	$-1.0 \cdot 10^{-2}$	$6.3 \cdot 10^{-2}$	$4.4 \cdot 10^{-3}$	$5.8 \cdot 10^{-1}$	$-2.7 \cdot 10^{-6}$	$-1.5 \cdot 10^{-6}$	$-2.0 \cdot 10^{-7}$	0.26 (-35)
7.a	Meuse fall 2021	$2.0 \cdot 10^{-7}$	$-1.5 \cdot 10^{-2}$	$8.9 \cdot 10^{-3}$	$1.9 \cdot 10^{-3}$	$3.5 \cdot 10^{-1}$	$-2.7 \cdot 10^{-4}$	$-4.4 \cdot 10^{0}$	$-1.4 \cdot 10^{-6}$	$1.9 \cdot 10^{-6}$	$-2.0 \cdot 10^{-7}$	0.34 (-279)
9.a	Meuse fall 2022	$6.7 \cdot 10^{-6}$	$-8.2 \cdot 10^{-3}$	$6.4 \cdot 10^{-3}$	$6.6 \cdot 10^{-3}$	$-5.5 \cdot 10^{-2}$	$1.1 \cdot 10^{-3}$	$-5.4 \cdot 10^{0}$	$-8.0 \cdot 10^{-7}$	$2.0 \cdot 10^{-6}$	$-9.0 \cdot 10^{-7}$	0.21 (-265)
10.a	IJssel winter 2024	$2.7 \cdot 10^{-3}$	$1.5 \cdot 10^{0}$	$2.2 \cdot 10^{-1}$	$3.1 \cdot 10^{-1}$	$-1.5 \cdot 10^{1}$	$-4.7 \cdot 10^{-2}$	$1.6 \cdot 10^{3}$	$9.6 \cdot 10^{-5}$	$2.0 \cdot 10^{-6}$	$-1.5 \cdot 10^{-4}$	0.83 (57)
10.b	IJssel winter 2024	$2.6 \cdot 10^{-3}$	$1.9 \cdot 10^{0}$	$3.2 \cdot 10^{-1}$	$2.3 \cdot 10^{-1}$	$-1.3 \cdot 10^{1}$	$-3.2 \cdot 10^{-2}$	$9.5 \cdot 10^{2}$	$5.8 \cdot 10^{-5}$	$-1.8 \cdot 10^{-5}$	$6.6 \cdot 10^{0}$ (*)	0.80 (63)
10.c	IJssel winter 2024	$2.7 \cdot 10^{-3}$	$1.7 \cdot 10^{0}$		$3.2 \cdot 10^{-1}$	$-1.5 \cdot 10^{1}$	$-4.6 \cdot 10^{-2}$	$1.6 \cdot 10^{3}$	$9.6 \cdot 10^{-5}$		$-1.5 \cdot 10^{-4}$	0.83 (53)
11.a	IJssel winter 2021	$2.9 \cdot 10^{-5}$	$-9.8 \cdot 10^{-2}$	$1.1 \cdot 10^{-1}$	$4.6 \cdot 10^{-3}$	$-1.3 \cdot 10^{-1}$	$2.5 \cdot 10^{-3}$	$9.6 \cdot 10^{0}$	$-5.9 \cdot 10^{-6}$	$-1.3 \cdot 10^{-6}$	$2.0 \cdot 10^{-6}$	0.24 (-183)
12.a	IJssel fall 2020	$-1.4 \cdot 10^{-5}$	$1.9 \cdot 10^{-2}$	$-4.1 \cdot 10^{-2}$	$1.0 \cdot 10^{-3}$	$1.7 \cdot 10^{-1}$	$-1.2 \cdot 10^{-3}$	$1.1 \cdot 10^{1}$	$7.0 \cdot 10^{-7}$	$-1.4 \cdot 10^{-6}$	$-1.4 \cdot 10^{-6}$	0.37 (-115)
13.a	IJssel fall 2021	$-4.4 \cdot 10^{-6}$	$-3.0 \cdot 10^{-3}$	$3.0 \cdot 10^{-3}$	$2.1 \cdot 10^{-3}$	$-5.9 \cdot 10^{-3}$	$8.3 \cdot 10^{-5}$	$1.1 \cdot 10^{0}$	$6.0 \cdot 10^{-7}$	$-7.7 \cdot 10^{-9}$	$-1.2 \cdot 10^{-6}$	0.26 (-357)
14.a	IJssel fall 2022	$4.7 \cdot 10^{-6}$	$3.4 \cdot 10^{-2}$	$-5.5 \cdot 10^{-2}$	$2.0 \cdot 10^{-2}$	$1.2 \cdot 10^{0}$	$2.0 \cdot 10^{-3}$	$-3.7 \cdot 10^{1}$	$-7.0 \cdot 10^{-7}$	$2.4 \cdot 10^{-6}$	$4.0 \cdot 10^{-7}$	0.30 (-183)
15.a	IJssel fall 2023	$-9.0 \cdot 10^{-7}$	$1.6 \cdot 10^{-2}$	$-1.5 \cdot 10^{-2}$	$7.7 \cdot 10^{-3}$	$-1.5 \cdot 10^{-2}$	$5.1 \cdot 10^{-4}$	$-2.3 \cdot 10^{0}$	$-5.0 \cdot 10^{-7}$	$-3.0 \cdot 10^{-7}$	$3.0 \cdot 10^{-7}$	0.43 (-235)

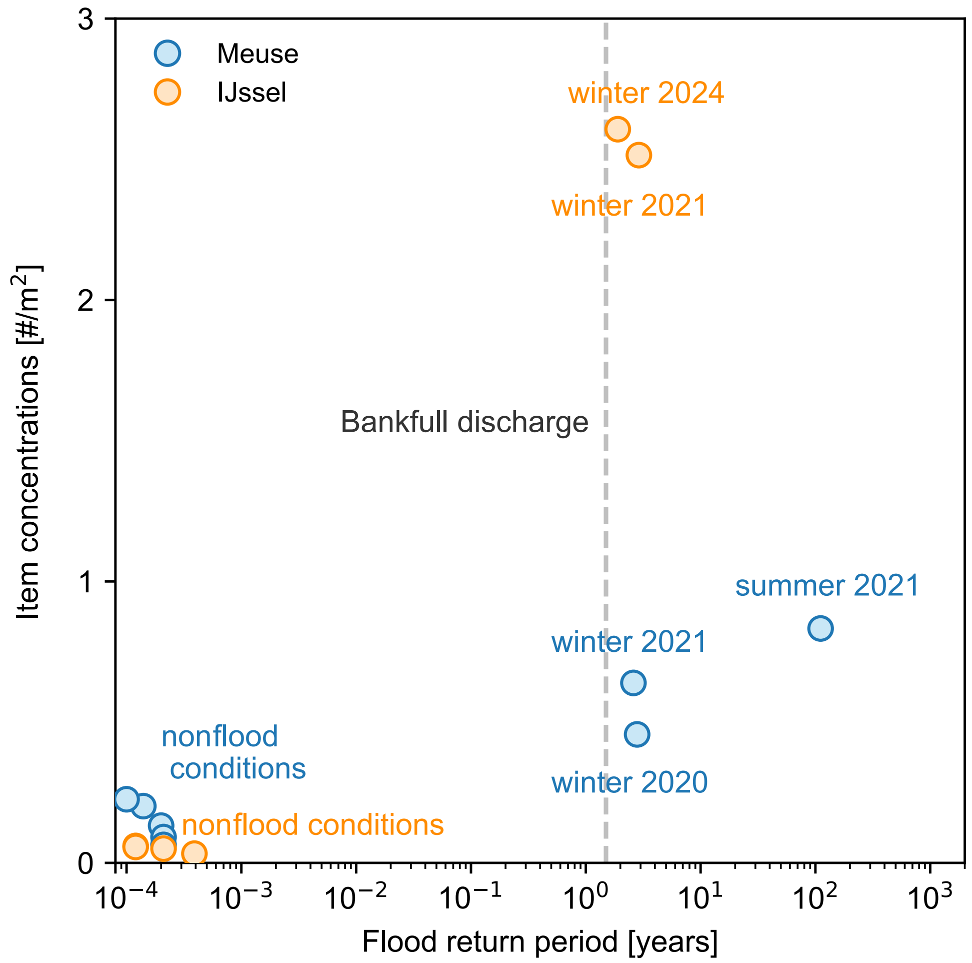

Figure S1: Macroplastic item concentrations increase as a function of flood return period. A steeper increase is noticeable for the IJssel compared to the Meuse, suggesting that the IJssel experiences higher rates of fragmentation and/or mobilization of sources containing numerous small macroplastic items as a result of floods. The bankfull discharge is indicated for the 1.5-year return period, consistent with established literature on bankfull discharge in natural rivers[1,2].

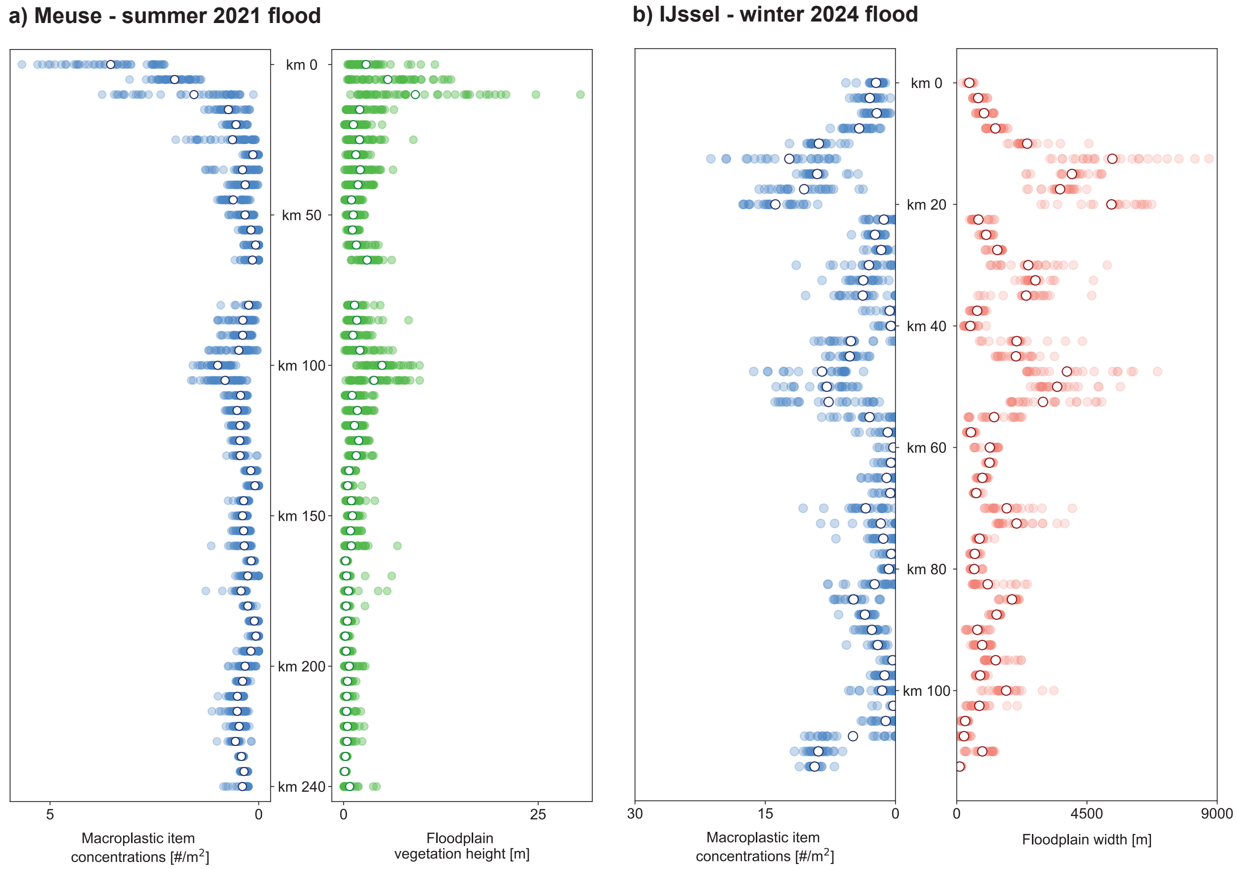

Figure S2: Modeled macroplastic concentrations along the upstream-downstream gradient, with key explanatory variables for the Meuse during the summer 2021 flood (a) and the IJssel during the winter 2024 flood (b). All values were binned at 5-km resolution for the Meuse and 2.5 km resolution for the IJssel. White dots represent mean values, while colored dots show individual data points.

References

(1) Dury, G.; Hails, J.; Robbie, H. Bankfull discharge and the magnitude frequency series. *Australian Journal of Science* **1963**, *26*, 123–124.

(2) Leopold, L. B.; Wolman, M. G.; Miller, J. P.; Wohl, E. E. *Fluvial processes in geomorphology*; Courier Dover Publications, 1964; p 544.
